# Natural enemies of herbivores maintain their biological control potential under short‐term exposure to future CO_2_, temperature, and precipitation patterns

**DOI:** 10.1002/ece3.7314

**Published:** 2021-03-16

**Authors:** Cong van Doan, Marc Pfander, Anouk S. Guyer, Xi Zhang, Corina Maurer, Christelle A.M. Robert

**Affiliations:** ^1^ Institute of Plant Sciences University of Bern Bern Switzerland; ^2^ Oeschger Centre for Climate Change Research (OCCR) University of Bern Bern Switzerland; ^3^ Present address: Agroscope Wädenswil Switzerland; ^4^ Present address: Key Laboratory of Plant Stress Biology State Key Laboratory of Cotton Biology School of Life Sciences Henan University Kaifeng China; ^5^ Present address: Agroecology and Environment Agroscope Zürich Switzerland

**Keywords:** climate change, herbivore natural enemies, nematodes, parasitoids, predators, trophic interactions

## Abstract

Climate change will profoundly alter the physiology and ecology of plants, insect herbivores, and their natural enemies, resulting in strong effects on multitrophic interactions. Yet, manipulative studies that investigate the direct combined impacts of changes in CO_2_, temperature, and precipitation on the third trophic level remain rare. Here, we assessed how exposure to elevated CO_2_, increased temperature, and decreased precipitation directly affect the performance and predation success of species from four major groups of herbivore natural enemies: an entomopathogenic nematode, a wolf spider, a ladybug, and a parasitoid wasp. A four‐day exposure to future climatic conditions (RCP 8.5), entailing a 28% decrease in precipitation, a 3.4°C raise in temperature, and a 400 ppm increase in CO_2_ levels, slightly reduced the survival of entomopathogenic nematodes, but had no effect on the survival of other species. Predation success was not negatively affected in any of the tested species, but it was even increased for wolf spiders and entomopathogenic nematodes. Factorial manipulation of climate variables revealed a positive effect of reduced soil moisture on nematode infectivity, but not of increased temperature or elevated CO_2_. These results suggest that natural enemies of herbivores may be well adapted to short‐term changes in climatic conditions. These findings provide mechanistic insights that will inform future efforts to disentangle the complex interplay of biotic and abiotic factors that drive climate‐dependent changes in multitrophic interaction networks.

## Graphical abstract

While herbivore enemies shape ecosystem functioning and biodiversity, their response to climate change remains unclear. By simultaneously controlling temperature, CO_2_, and precipitation levels, we assessed how future climate conditions directly affect the performance and predation success of four herbivore natural enemies. We found that short‐term exposure to projected climatic conditions does not impair the overall feeding efficiency of herbivore natural enemies but may lead to shifts in their foraging strategies.

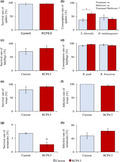

## 1 INTRODUCTION

Climate change entails increasing concentrations of greenhouse gases in the atmosphere, rising temperatures, and shifts in precipitation patterns (IPCC, 2014). These changes will profoundly alter individual species’ physiologies and ecology, interaction networks, community composition, and (agro)ecosystem functioning (Abdala‐Roberts et al., 2019; Bellard et al., 2012; Boukal et al., 2019; Newman et al., 2011; Parmesan & Yohe, 2003; Selvaraj et al., 2013; Walther, 2010). The feeding efficiency of natural enemies of herbivores in particular was predicted to be altered by climate change (Rosenblatt & Schmitz, 2016; Tylianakis et al., 2008; Voigt et al., 2003), which may lead to strong effects on trophic interaction networks and biological control services (Altieri et al., 2012; Bohan et al., 2013; Gravel et al., 2016; van der Putten et al., 2004; Vidal & Murphy, 2018; Woodward & Bohan, 2013). Yet, manipulative studies that investigate the combined impacts of changes in CO_2_, temperature, and precipitation on members of higher trophic levels remain rare (Jamieson et al., 2012; Laws, 2017; Rosenblatt & Schmitz, 2016), thus limiting our capacity to predict how climate change will affect natural enemies of herbivores.

Natural enemies of herbivores such as predators, parasites, and parasitoids may suffer from climate change via extrinsic and intrinsic mechanisms. Intrinsic mechanisms refer to direct effects of climate‐associated abiotic parameters on an organism (Cornelissen, 2011; Robinet & Roques, 2010). Extrinsic mechanisms refer to indirect effects, mediated for instance through shifted distributions, altered phenology or physiology of herbivores and their host plants (Chidawanyika et al., 2019; Damien & Tougeron, 2019; Han et al., 2019; Jeffs & Lewis, 2013; Kaplan et al., 2016; Kharouba et al., 2018; Pincebourde et al., 2017; Renner & Zohner, 2018). While investigating indirect effects of climate change across trophic levels is crucial to predict community shifts (van der Putten et al., 2010), understanding the direct impact of climate change onto each trophic level is essential to characterize the underlying mechanisms (Thomson et al., 2010).

Over the last years, detailed experiments have been conducted to evaluate the direct impact of individual abiotic factors related to climate change on natural enemies of herbivores. For example, temperature was identified as a key determinant of parasitoid developmental rates, survival, fecundity, parasitism, and dispersal (Hance et al., 2007; Selvaraj et al., 2013; Walther et al., 2002). Thermal performance curves revealed that increasing temperatures enhance parasitism success until a maximum at optimum temperature, beyond which any increase in temperature leads to a decline of parasitism success (Chidawanyika et al., 2019; Furlong & Zalucki, 2017). Optimum temperature was repeatedly reported to be lower for parasitoids than for their hosts, indicating that parasitoids may be more susceptible to elevated temperature than herbivores, resulting in lowered biocontrol efficacy over time (Furlong & Zalucki, 2017). Similarly, changes in CO_2_ levels and precipitation were demonstrated to affect natural enemies of herbivores. While the impact of elevated CO_2_ on the third level is generally mediated by shifts in bottom‐up forces, direct effects of CO_2_ on predators and parasitoids should not be neglected (Jun Chen et al., 2007). Notably, elevated CO_2_ alters the chemosensation of natural enemies by disrupting their ability to perceive or process cues from their environment (Draper & Weissburg, 2019), for instance, through a reduction of olfactory receptor sensitivity and habituation of higher‐order neurons (Majeed et al., 2014). Changes in precipitation were reported to disturb the physiology and behavior of herbivore natural enemies (Barnett & Facey, 2016; Jamieson et al., 2012; Torode et al., 2016). While arachnids and insects possessing a waxy cuticle can reduce evaporation (Berridge, 2012), soft body organisms (nematodes, isopods, and myriapods) may suffer larger water loss under drought conditions (Sylvain et al., 2014). Drought may also prompt arthropod predators to migrate, hide in the soil, or build shelters to avoid desiccation (Berridge, 2012; Melguizo‐Ruiz et al., 2016; Willmer, 1982), thus reducing their foraging activity.

Compared to single climate parameters, much less is known about the direct effects of combined and interactive impact of multiple parameters on natural enemies of herbivores (Hiltpold et al., 2016; Jactel et al., 2019; Kreyling & Beier, 2013; Robinson et al., 2012). A meta‐analysis on the impact of multiple global change drivers on the survival of animals from all trophic levels revealed that combined climatic stressors led to nonadditive effects, comprising synergistic and antagonist interactions, in the majority (65%) of the examined studies (Darling & Côté, 2008). The occurrence of synergistic and antagonist effects varies considerably between meta‐analyses and is species and sex‐dependent (Crain et al., 2008; Darling & Côté, 2008; Rosenblatt & Schmitz, 2014). Studies evaluating the direct effects of combined climatic factors on herbivore natural enemies remain scarce. One example showed antagonist interactions between elevated temperature and decreased precipitation pattern on parasitoid wasp success: While elevated temperature and drought individually enhanced parasitism success, their combination reversed this effect (Romo & Tylianakis, 2013). Another study, using altitudinal gradient experiments, revealed that warming, elevated nitrogen resources, and their combination resulted in similar increase in total herbivore biomass, but not that of parasitoids (Sassi & Tylianakis, 2012). Even though parasitoids parasitized more herbivores under elevated temperature, this increase was not proportionate to the increase in host abundance. This observation could be explained by the negative effect of both drivers and their subadditive interactions on parasitism rates (Sassi & Tylianakis, 2012). Despite these examples, to date, the outcome of multiple global change factors on the third trophic level remains unpredictable and is likely to lead to “ecological surprises” (Darling & Côté, 2008).

Here, we developed a microcosm climate system to quantify the direct impact of elevated temperature (incl. an appropriate diurnal rhytm) and CO_2_ as well as reduced precipitation, as predicted by the Representative Concentration Pathway 8.5 (RCP 8.5, (IPCC, 2014)), on the survival and foraging efficiency of different natural enemies of herbivores. Focusing on direct effects comes with the limitation that no preys can be inserted in the system during exposure. Our study therefore investigated the short‐term impact of climate change on herbivore enemies. We reveal that exposure to RCP 8.5 conditions has no negative direct effects on several different natural enemies of herbivores, suggesting that the third trophic level may be resilient to direct abiotic effects imposed by climate change.

## 2 MATERIALS AND METHODS

### 2.1 Biological resources

*Herbivores*. Eggs of the cucumber banded beetle, *Diabrotica balteata* (Coleoptera: Chrysomelidae), were kindly provided by Oliver Kindler (Syngenta, Stein, Switzerland). Hatching larvae were reared on freshly germinated maize seedlings (*var*. Akku, DSP, Delley, Switzerland) until use. Third instar larvae were used for all infectivity experiments. Eggs of Egyptian cotton leafworm, *Spodoptera littoralis* (Lepidoptera: Noctuidae), were provided by Ted Turlings (Laboratory of Fundamental Research in Fundamental and Applied Chemical Ecology, University of Neuchâtel, Neuchâtel, Switzerland) and reared on artificial diet until use. Larvae of the common fruit fly, *Drosophila melanogaster* (Diptera: Drosophilidae), were provided by Dirk Beuchle (Group Suter, Institute of Cell Biology, University of Bern, Bern, Switzerland). Individuals of the bird cherry‐oat aphids, *Rhopalosiphum padi* (Hemiptera: Aphididae), were bought from Andermatt Biocontrol (Grossdietwil, Switzerland) and reared on barley until use. Individuals of the cabbage aphid, *Brevicoryne brassicae* (Hemiptera: Aphididae), were collected in a cabbage field in Zürich (47°22′17.40″ N/8°67′50.18″ E, Agroscope in Wädenswil, Zürich, Switzerland).

*Predators and parasites*. Ladybirds, *Adalia bipunctata* (Coleoptera: Coccinellidae), and parasitoid wasps, *Aphidius ervi* (Hymenoptera: Aphidiidae), were bought from Andermatt Biocontrol (Grossdietwil, Switzerland). Wolf spiders, *Alopecosa albofasciata* (Araneae: Lycosidae), were collected in the field (Coordinates: 46°97′49.2″ N/7°43′77.4″ E, Bremgarten bei Bern, Switzerland). Entomopathogenic nematodes (EPNs), *Heterorhabditis bacteriophora*, strains EN01, TT01, DE6, HU2 and PT1 were obtained from in‐house colonies (Zhang et al., 2019). All EPN strains were reared in larvae of the wax moth, *Galleria mellonella* (Lepidoptera: Pyralidae) (Fischereibedarf, Bern, Switzerland), until use (McMullen II & Stock, 2014). Infective juveniles (IJs) were collected in aqueous suspension from white traps (White, 1927) in tap water (1 EPN/µL) at room temperature for 10 days before use.

### 2.2 Current and predicted climatic conditions

Current and predicted climatic conditions were calculated using climatic data from the Swiss Central Plateau (Average of summer conditions from 2004 to 2016, Oensingen, 47°17′11.1″ N/7°44′01.5″ E, Switzerland), data were supported by MeteoSwiss (Federal Office of Meteorology and Climatology, Zürich, Switzerland), and predictions from the Representative Concentration Pathway 8.5 (RCP 8.5, Intergovernmental Panel on Climate Change (IPCC) report (IPCC, 2014). RCP 8.5 corresponds to an extreme scenario in which CO_2_ emissions continue to rise throughout the 21st century. Consequently, current and RCP 8.5 atmospheric CO_2_ concentrations were of 450 ppm (±50 ppm), and 850 ppm (±50 ppm), respectively. Current and RCP 8.5 of soil temperatures were 19.6°C and 23°C, respectively. Because daily temperature variation can affect insect performance and predator–prey interactions (Stoks et al., 2017), current and RCP 8.5 soil temperatures followed a diurnal variation of 3.5°C (minimal temperature at 6 a.m. and maximal temperature at 4 p.m.) and reached a maximum temperature of 21.4°C and 24.8°C, respectively (Figure S1). Current and RCP 8.5 soil volumetric moisture levels were adjusted to 23% and 16.6% (corresponding to 28% less precipitation (Figure S2)).

### 2.3 Microcosm systems

To manipulate CO_2_ levels, temperature, and moisture, we developed a microcosm system using dry‐bath cyclers and a custom‐made CO_2_‐dosage system. A detailed description of the microcosms can be found in Supplementary Material (Material S1). Falcon tubes (50 ml, Falcon, Greiner Bio‐One, Frickenhausen, Germany) were filled with 30 g dry (80°C for 48 hr), sieved (2 cm mesh) soil (40% sand, 35% silt, 25% clay; Landerde, Ricoter, Aarberg, Switzerland). The natural soil microbiota was re‐implemented to the soil as previously described (Hu et al., 2018). All falcon tubes were placed in dry‐bath cyclers (Digital Heating Cooling Drybath, Thermo Scientific, Fisher Scientific AG, Reinach, Switzerland). Ambient air from outside the building was used to mimic current conditions (=450 ppm ± 50 ppm). A concentration of 400 ppm CO_2_ (purity 100%, 54.6 L bottle, and pressure of output at 0.8 bars, Gümligen, Switzerland) was added to ambient air (=850 ppm ± 50 ppm) to reach expected RCP 8.5 scenarios. The resulting CO_2_: air mix was pushed through a filter of activated carbon (Camozzi, Warwickshire, United Kingdom). The flow rate sent through individual Falcon tubes was adjusted to 1 L/min. The outflow of the Falcon tubes was connected to a collection system, and a CO_2_ sensor to verify CO_2_ levels. The collected air was then released in the environment.

The temperature in the Falcon tubes was controlled through the dry‐bath cyclers and followed a diurnal variation of 3.5°C. Soil temperatures used to mimic current conditions were of 17.8°C at 6 a.m., and gradually increased to reach 21.4°C at 4 p.m. (Figure S1), as reported for the Swiss Plateau over the past two decades (MeteoSwiss, Federal Office of Meteorology and Climatology, Zürich, Switzerland). The temperatures mimicking the RCP 8.5 scenario were set to 21.2°C at 6 a.m. and progressively increased to reach 24.8°C at 4 p.m. (Figure S1).

The moisture present in the tubes was controlled by adding the soil leachates to the tubes once at the beginning of the experiment. The volume of water to add in the tubes was calculated based on the soil density of 1.2 g/cm^3^. Current moisture levels (23% soil moisture) were achieved by adding 16.6% (v/v) microbiota extracts contained in tap water and 6.4% (v/v) additional tap water. Predicted moisture levels (RCP 8.5, 28% less precipitation, Figure S2) were achieved by adding 16.6% (v/v) microbiota extracts contained in tap water only. The temperatures were adjusted to the different scenarios over a six‐hour adaptation period (Figure S1). The water loss over the run of the experiments was not significantly different between treatments.

### 2.4 Effects of climate change on aboveground predator and parasite survival

To evaluate the direct impact of current and predicted climatic conditions on success of predators, we evaluated the survival and predation success, defined as foraging efficiency, of a wolf spider (*A. albofasciata*, *n* = 25–30), a ladybug (*A. bipunctata*, *n* = 17), and a parasitic wasp (*A. ervi*, *n* = 5–6) after exposure to climatic conditions in the microcosms. Half of the individuals for each predator species were exposed to current conditions, while the other half was exposed to RCP 8.5 conditions. A piece of cotton soaked in 10% sucrose solution, or a slice of apple was added to the tubes as a sugar source. After three days of incubation, the temperature in all systems ramped to ambient conditions to prevent any temperature‐related physiological shock. All herbivore enemies were exposed to ambient temperature levels for one day prior predation assays. This resulted in exposure to current and predicted temperature for three days, and to current or predicted CO_2_ and precipitation levels for 4 days prior predation assays. This four‐day duration chosen based on the assumption it would be sufficient to trigger any possible impact on the organisms’ physiologies without leading to lethal starvation. All predation assays were performed under ambient conditions. To measure direct effects of climate change only, all herbivores were kept in ambient conditions at all time.

### 2.5 Effects of climate change on aboveground predation and parasitism success

To assess the predation and parasitism success of the three aboveground arthropods, four independent assays were conducted as follows. In a first experiment, individual wolf spiders were exposed to current or predicted RCP 8.5 climatic conditions as described above. After exposure, each spider was placed in a solo cup (250 ml, Pack Markt Sabaratnam, Solothurn, Switzerland). Half of the cups contained ten second instar *S. littoralis* caterpillar larvae, while the second half contained ten *D. melanogaster* flies (*n* = 9–12). In a second assay, individual ladybugs were exposed to current or RCP 8.5 climatic conditions (*n* = 9–14) and then placed in Petri dishes (94 x 16 mm, Bio‐One Petri Greiner, Huberlab, Switzerland) containing a piece of cotton soaked in a 10% sucrose solution and thirty aphids *R. padi*. In the third experiment, three ladybugs per tube were exposed to current or predicted RCP 8.5 conditions (*n* = 5–6) and then placed in Petri dishes containing a piece of cotton soaked in a 10% sucrose solution and thirty aphids *B. brassicae*. In these assays, the number of consumed preys was recorded 24 hr after introduction of the predator. In a fourth assay, nine to twelve parasitoid wasps were placed in each tube and exposed to either current or predicted RCP 8.5 conditions (*n* = 5–6). The wasps were then placed into Petri dishes containing a slice of apple and thirty aphids *B. brassicae*. The wasps and aphids were left together for five days before assessing the aphid parasitism rate.

### 2.6 Climate change on belowground parasitism rates

To investigate direct effects of an extreme climate change scenario (RCP 8.5) on the ability of soil‐dwelling parasites to infect an herbivore, five strains of the entomopathogenic nematode *H. bacteriophora* (strains EN01, TT01, DE6, HU2, PT1) were tested in the microcosms described above. Briefly, 3,000 infective juvenile nematodes, suspended in one mL tap water, were added in each microcosm, and exposed to current or RCP 8.5 climatic conditions for three days. After a one‐day incubation period to ambient temperature levels, the soil from the microcosms was collected and poured into a plastic cup (250 ml). The soil moisture was then adjusted to 23% in all treatments. Ten third instar larvae of the root herbivore *D. balteata* were added into each plastic cup. After seven days, all larvae were placed in white traps (White, 1927). The parasitism rate and number of emerging juveniles were recorded after 14 days. The number of EPN emerging from larvae infected with EPNs originating from one tube was averaged and used as a proxy for EPN fitness. Testing five strains with sufficient replicate numbers required four independent experiments, each including EN01 and two additional strains (*n* = 7–16 per strain and treatment in total). The infectivity and number of EPNs emerging from the insect cadavers were expressed relatively to EN01‐ambient conditions treatment.

### 2.7 Effects of single climatic conditions on EPN performance and fitness

To appraise the individual and interactive effects of soil temperature, soil moisture, and CO_2_ onto EPN performance and fitness, a full factorial experiment was designed, using all combinations between current and predicted climatic variable levels. The assays were performed using the nematode *H. bacteriophora* strain EN01 and following the same methods as described above. In a first assay, EPN survival was recorded by collecting EPNs using a modified Baermann funnel method (Baermann, 1917). Briefly, soil samples were placed into a paper towel and rehydrated until saturation in a funnel, which was sealed with metal clips at the bottom. EPN extraction was performed at ambient temperature conditions. Extracts with suspended EPNs were collected after 24 and 48 hr. The proportion of EPNs collected after 24 hr over the total recovered EPN number was used as a proxy for mobility. In a second assay, EPNs were collected and used for infectivity and fitness assessment as described above. This experiment was repeated three times to ensure good replicate numbers (*n* = 6–12 per treatment).

### 2.8 Statistical analyses

Statistical analyses were conducted using R (version 3.5.3, https://www.r‐project.org) and online tools (http://quantpsy.org; https://www.graphpad.com). Normality and heteroscedasticity of error variance were assessed using Levene's and Shapiro–Wilk tests, as well as by visualizing quantile–quantile plots and model residuals versus fitted values. The survival rates of spiders and ladybugs were analyzed using chi‐squared tests incorporating a Yates correction for continuity. The survival rate of wasps was analyzed using a generalized linear model using the proportion of surviving wasps in each tube as a response to climatic scenarios. Spider predation rates were analyzed using linear models. Ladybug predation and wasp parasitism rates were analyzed using generalized linear models with a binomial error structure. EPN infectivity and emergence data were expressed relatively to the EN01 strain in ambient conditions. EPN emergence data from insect infected with EPNs originating from the same microcosm tube were averaged and ln transformed prior analysis. One emergence data point was considered as outlier (Grubbs’ test, *p* <.05) and removed from the analysis. EPN relative infectivity and fitness were analyzed using linear models. Climatic scenarios/variables, herbivore species (when compared within a single experiment), and nematode strains were treated as fixed effects. Cyclers and CO_2_ channels did not increase the model gain on likelihood and were therefore removed from the models. Experimental repetitions were treated as fixed variables. Significant models were evaluated by Tukey Honest Significant Differences (TukeyHSD) post hoc tests.

## 3 RESULTS

### 3.1 Predicted climatic conditions do not directly influence the survival of aboveground predators

**FIGURE 1:**
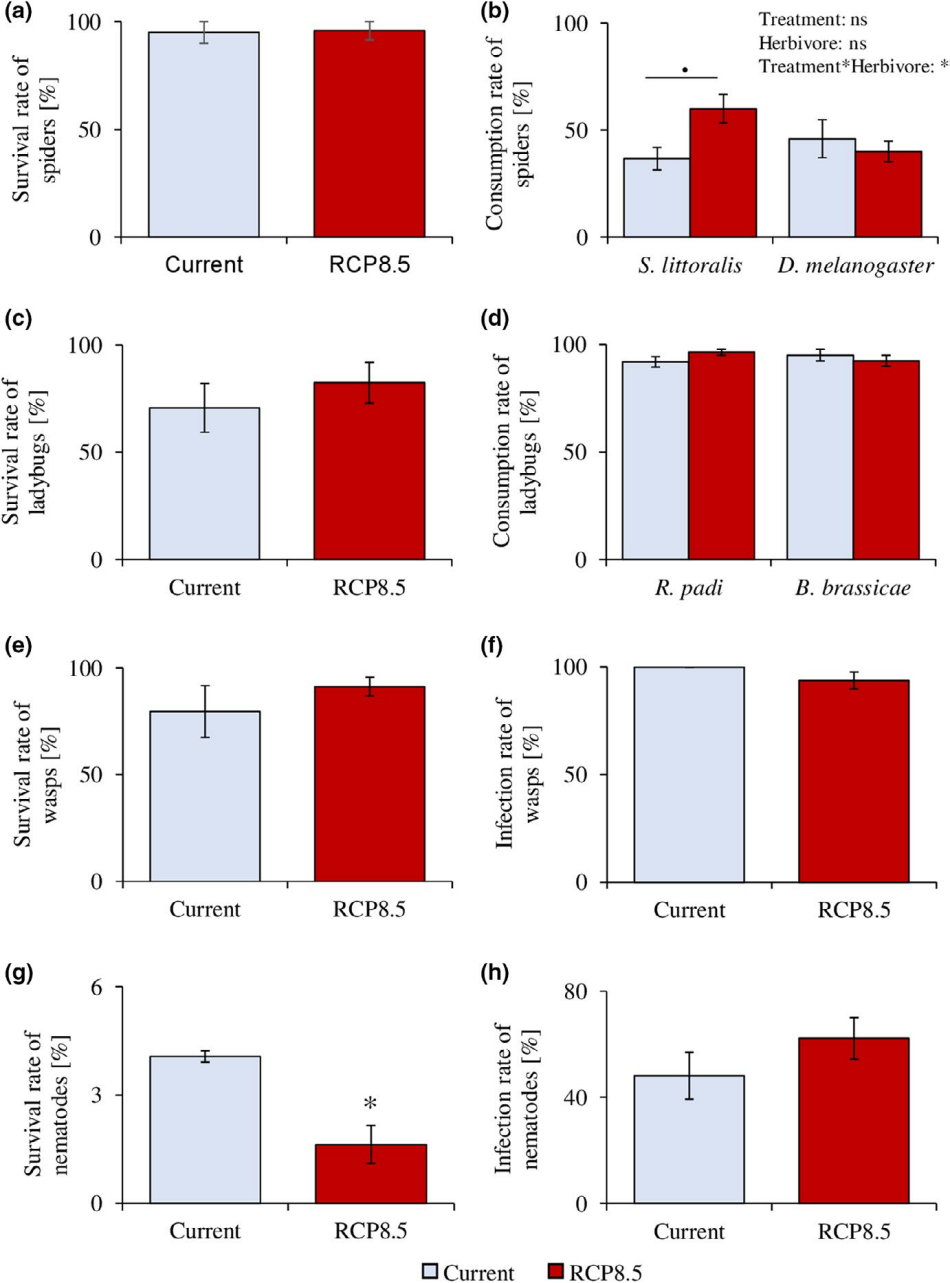
Survival and predation efficacy of herbivore natural enemies after exposure to current and predicted RCP 8.5 climate scenario (IPCC, 2014). Survival (Yates’ Chi square, mean ± se, *n* = 20–24) and consumption rate (linear models, means ± se, *n* = 9–12) of the wolf spider, *Alopecosa albofasciata*, after exposure to temperature, precipitation and CO_2_ levels of current or predicted (RCP 8.5) climate scenarios (a, b). Survival (Yates’ Chi‐square, means ± se, *n* = 17) and consumption rate (generalized linear models, means ± se, *n* = 6–14) of the ladybird, *Adalia bipunctata*, after exposure to temperature, precipitation, and CO_2_ levels of current or predicted (RCP 8.5) climate scenarios (c, d). Survival (generalized linear model, means ± se, *n* = 5–6) and infection rate (generalized linear models, means ± se, *n* = 5–6) of the parasite wasp, *Aphidius ervi*, after exposure to temperature, precipitations, and CO_2_ levels of current or predicted (RCP 8.5) climate scenarios (e, f). Survival rate (generalized linear model, means ± se, *n* = 2–3) and infection rate (generalized linear models, means ± se, *n* = 6–9) of the commercial entomopathogenic nematode, strain EN01, under exposure to temperature, moistures and CO_2_ corresponding to current or predicted (RCP 8.5) climate scenarios (g, h). Stars indicate significant differences, *: *p* <.05. Dots indicate trends,.: 0.05 < *p* <.10

The designed microcosms ensured good survival rates of wolf spiders, ladybugs, and parasitoid wasps over the course of the experiment (Figure 1). Exposure to RCP 8.5 climate conditions, including elevated levels of CO_2_, elevated temperature, and decreased moisture, did not impair the survival of any of the tested arthropods (Figure 1a, c, e).

### 3.2 Predicted climatic conditions altered aboveground predation in a species‐specific manner

Exposure to current or predicted RCP 8.5 climatic conditions resulted in species‐specific changes in predation success. The combination of elevated CO_2_, increased temperature, and decreased moisture (RCP 8.5) altered the spider predation rates in a prey‐specific manner. It slightly increased spider consumption of the cotton leafworm, *S. littoralis*, but not of the fly larvae, *D. melanogaster* (Figure 1b). Exposure of ladybugs to current or predicted RCP 8.5 climatic conditions resulted in similar consumption rates of cabbage‐ and bird cherry‐oat aphids (Figure 1d). Similarly, exposure to current or predicted RCP 8.5 climatic conditions did not alter the wasp parasitism rate of cabbage aphids (Figure 1f).

### 3.3 Predicted climatic conditions increase herbivore infection rates by nematodes

**FIGURE 2:**
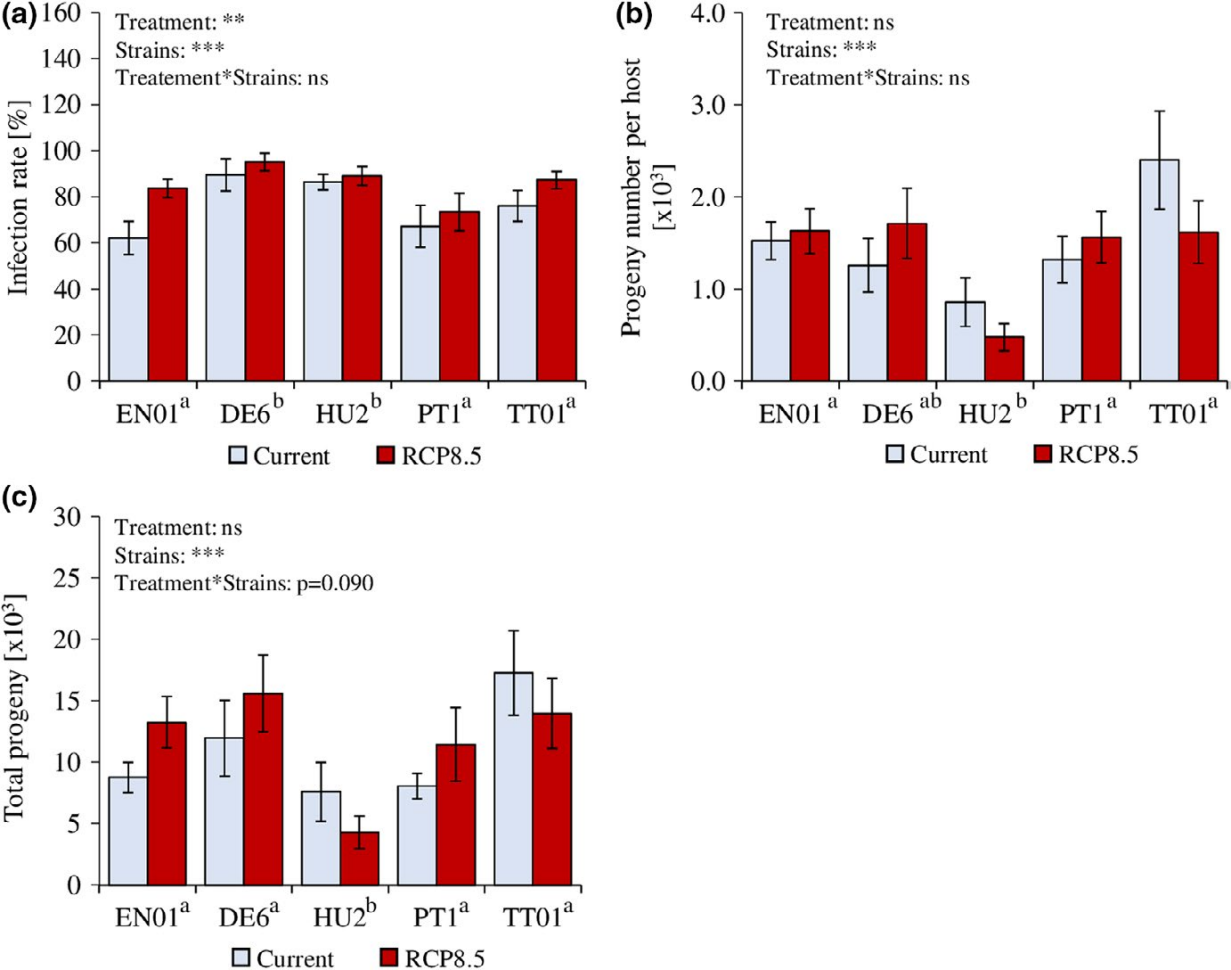
Entomopathogenic nematode infection rate and progeny numbers after exposure to current and predicted RCP 8.5 climate scenario (IPCC, 2014). Infection rate (generalized linear model, mean ± se, *n* = 7–16) of five different strains of the entomopathogenic nematode *H. bacteriophora* on *Diabrotica balteata* larvae after exposure to temperature, precipitations, and CO_2_ corresponding to current or predicted (RCP 8.5) climate scenarios (a). Number of infective juveniles (generalized linear model, mean ± se, *n* = 6–16) emerging per infected host cadaver in five strains (b). Total progeny number calculated as the product between the number of infective juveniles emerging per host and the number of infected hosts (generalized linear model, mean ± se, *n* = 6–12) (c). Stars indicate significant differences: ***: *p* ≤.001; **: *p* ≤.01; ns: nonsignificant. Different superscript letters next to the strain names indicate significant differences among strains

Direct exposure to current or predicted RCP 8.5 climatic conditions in microcosms did not decrease infection rates across five tested EPN strains (Figure 2a). On average, exposure to RCP 8.5 conditions increased EPN infection rates by 10.03%. The effect of the RCP 8.5 scenario was the strongest on EN01, whose infection rates increased by 21.44% compared to current conditions. Exposure to predicted RCP 8.5 scenarios did not alter the number of emerging juveniles per insect host in any of the tested strains, nor the overall progeny number (Figure 2b‐c).

### 3.4 Reduced soil moisture decreases EPN mobility, but increases EPN fitness

**FIGURE 3:**
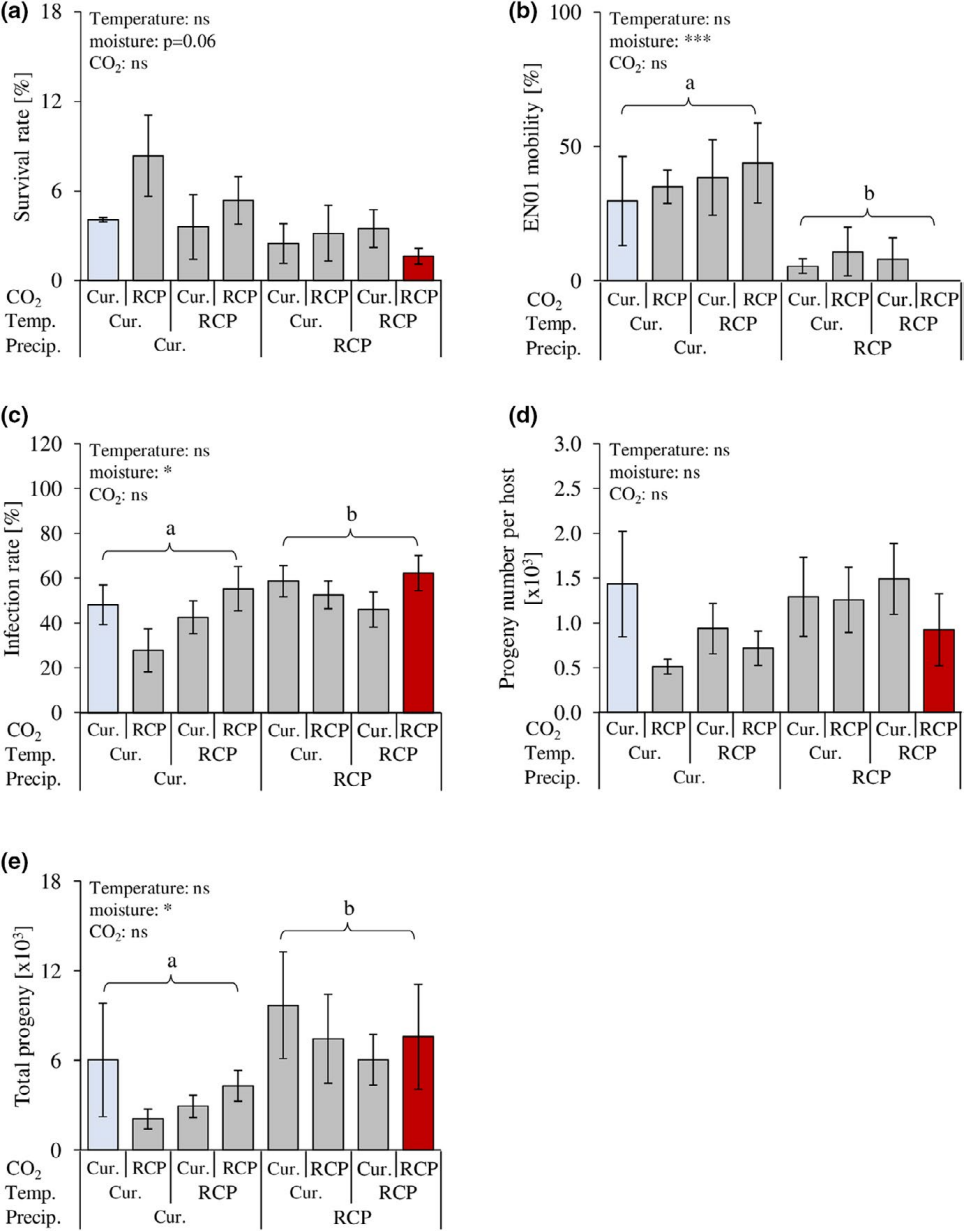
Effects of single and combined climatic variables on entomopathogenic nematode fitness and parasitism efficacy. Survival (linear model, Mean ± se, *n* = 2–4) of the entomopathogenic nematode (EPN), *Heterorhabditis bacteriophora* (strain EN01), after exposure to current and predicted RCP 8.5 temperature, precipitation, and CO_2_ (a). EPN mobility (generalized linear model, Mean ± se, *n* = 2–4) after exposure to current and predicted RCP 8.5 temperature, precipitation, and CO_2_ (b). EPN infection rate (generalized linear model, Mean ± se, *n* = 6–12) of the root herbivore *Diabrotica balteata* larvae after exposure to current and predicted RCP 8.5 temperature, precipitation, and CO_2_ (c). EPN progeny number (IJs) per infected *D. balteata* larva (linear model, Mean ± se, *n* = 4–11) after exposure to current and predicted RCP 8.5 temperature, precipitation, and CO_2_ (d). EPN total progeny (generalized linear model, Mean ± se, *n* = 4–11) after exposure to current and predicted RCP 8.5 temperature, precipitation, and CO_2_ (e). Temp.: temperature; Precip.: precipitation; Cur.: current, RCP: RCP 8.5 (IPCC, 2014). Stars indicate significant differences: ***: *p* ≤.001; *: *p* ≤.05; ns: nonsignificant. Different letters indicate significant differences. No interaction between the climatic variables was found to be significant

Factorial manipulation of the individual climatic variables revealed that exposure to lower soil moisture levels (−28% precipitation) modulated EPN success, while exposure to increased temperature and elevated CO_2_ levels had no additional effects. Reduced soil moisture slightly decreased EPN survival (albeit not significantly), and mobility (Figure 3a‐b), but increased infection rates (Figure 3c). While the impact of soil moisture on the progeny number per host was negligible (Figure 3d), the overall EPN fitness was increased (Figure 3e).

## 4 DISCUSSION

Our study highlights that a short exposure to temperature, CO_2,_ and precipitation levels predicted under an extreme climatic scenario (RCP 8.5) does not directly impair the efficacy of different natural enemies of herbivores, including several important biological control agents. We even observed a prey species‐specific increase in spider efficiency and enhanced nematode infection rates. This finding suggests that the negative impact of climate change on higher trophic levels observed in large meta‐analyses and field experiments may mostly be mediated through either long‐term exposure and/or indirect (plant and herbivore‐mediated) changes. The possible underlying mechanisms and ecological relevance of these observations are discussed below.

Current models predict that climate change will strongly harm higher trophic levels (Thakur, 2020; Voigt et al., 2003). Our study reveals that short‐term climatic‐related changes have negligible direct effects on the ability of third trophic level organisms to predate or parasitize herbivores. The negative impact of climate change on higher trophic levels observed in meta‐analyses and field experiments may therefore be mediated through either long‐term exposure or indirect changes. Exposure to future climatic scenario did not affect the survival of aboveground herbivore natural enemies such as spiders, ladybugs, and parasitoid wasps but decreased the survival of belowground entomopathogenic nematodes (EPNs) within four days. This observation concurs with the hypothesis that arthropods, possessing a waxy cuticle that protects them from desiccation, may be more resistant to climate change than soft‐bodied organisms (Sylvain et al., 2014). More investigation would be required to confirm this observation. While arthropod predators and parasites are prevalent aboveground (Nyffeler & Birkhofer, 2017), EPNs are key herbivore enemies belowground (Denno et al., 2008; Půza & Mrácek, 2005). The contrasting impact of climate change on above‐ and belowground organisms survival supports the hypothesis that green, aboveground, food webs may be more sensitive to climate change than brown, belowground ones (Thakur, 2020). Yet, future climatic conditions did not negatively impact the predation or parasitism rates by herbivore natural enemies, and even increased the success of nematodes and spiders. The increased infection rate by nematodes was sufficient to compensate for their lower survival in term of progeny number. While climate change may not directly impair EPN ability to control herbivore pest population, changes in host abundance, phenology and quality can drastically modulate their efficacy (Guyer et al., 2018; Hiltpold et al., 2016, 2020; Půza & Mrácek, 2005). Interestingly, the increase in spider predation rate was prey species‐specific and may reflect changes in prey preferences. Such shift in prey consumption was previously observed in Arctic spiders, which ultimately led to slower decomposition rates in soil (Koltz et al., 2018). As spiders consume up to 800 million tons of prey per year worldwide (Nyffeler & Birkhofer, 2017), identifying future shifts in their predation strategy will be crucial to reliably predict changes in food webs and ecosystem functioning.

Understanding how different climatic variables act together to impact the efficiency of natural enemies of herbivores is an important frontier in multitrophic interaction ecology. Investigating the impact of single and combined climate‐related drivers onto EPN survival and efficacy revealed no interaction between elevated temperature, CO_2,_ and decreased precipitation. While temperature is known to be a major driver of EPN survival and infectivity (Aatif et al., 2020; Lalramliana & Yadav, 2016; Pervez et al., 2015), we did not find any impact of warming in our study. This discrepancy may be explained by the low range of temperature used in our study (19.6–23°C) compared to the described beneficial temperature range (25–30°C) (Pervez et al., 2015). Consistently with previous literature, elevated levels of CO_2_ did not affect EPN survival and parasitism (Hiltpold et al., 2020). Instead, lower precipitation alone seemed to contribute to increased infection rates and progeny number, while decreasing the nematode survival and mobility in the soil. Nematodes have evolved physiological and behavioral strategies to cope with low environmental moisture (Grewal et al., 2011; Kagimu et al., 2017). They can respond to desiccation by initiating anhydrobiosis, a phenomenon largely associated with the accumulation of trehalose and water stress‐related proteins (Grewal et al., 2011; Kagimu et al., 2017; Womersley, 1990). The tolerance to desiccation conferred by anhydrobiosis varies considerably between EPN species and strains (Grewal et al., 2002; Mukuka et al., 2008). Furthermore, EPNs can avoid desiccation through aggregative movement patterns (Ruan et al., 2018) and migration to deeper soil layers where moisture levels are higher (Salame & Glazer, 2015). It was also suggested that EPNs can avoid desiccation by remaining longer inside their hosts (Brown & Gaugler, 1997; Koppenhöfer et al., 1997; Půza & Mrácek, 2005, 2007). It is tempting to speculate that the higher infection rates and progeny numbers observed in our study upon predicted climatic scenario are the result of some EPN desiccation tolerance strategy.

Climate change is a multifactorial phenomenon that may affect multitrophic interactions through direct and indirect effects. Interactive effects of the global change drivers and cascading effects among food webs render predictions about the response of multitrophic challenging to draw. Our study complements current models and shows that combined elevated temperature, CO_2_ levels and decreased precipitation do not have a direct negative impact on the performance of third trophic level organisms upon short‐term exposure. Together with the current literature, this suggest that indirect, bottom‐up‐mediated, climate change effects may be stronger than direct effects on predator and parasite efficacy in controlling herbivore populations. Yet, our study highlighted possible shifts in prey species, while the latter were kept in ambient conditions. This phenomenon may have substantial repercussions on ecosystem functioning and would deserve more attention. Building reliable models of food web response to climate change will be of crucial importance to face future natural and agricultural challenges.

## CONFLICT OF INTEREST

The authors declare having no competing interests.

## AUTHOR CONTRIBUTIONS

**Cong van Doan:** Data curation (lead); Formal analysis (equal); Investigation (lead); Methodology (supporting); Visualization (equal); Writing‐original draft (lead); Writing‐review & editing (supporting). **Marc Pfander:** Methodology (equal); Software (lead); Writing‐original draft (supporting). **Anouk Sabine Guyer:** Conceptualization (supporting); Data curation (supporting); Investigation (supporting). **Xi Zhang:** Data curation (supporting); Investigation (supporting); Writing‐review & editing (supporting). **Corina Maurer:** Data curation (supporting); Investigation (supporting); Resources (supporting). **Christelle A.M. Robert:** Conceptualization (lead); Formal analysis (equal); Methodology (equal); Project administration (lead); Resources (lead); Supervision (lead); Visualization (equal); Writing‐original draft (supporting); Writing‐review & editing (lead).

## Supporting information

Material S1

Fig S1

Fig S2

Data S1

## Data Availability

All raw data are available in the supplementary material (Data S1).
