# Supplementary material for: Natural enemies of herbivores maintain their biological control potential under short‐term exposure to future CO2, temperature, and precipitation patterns
Source: Ecol Evol. 2021 Mar 16;11(9):4182–92. doi: 10.1002/ece3.7314 (PMC8093683; doi:10.1002/ece3.7314)

***Supplementary Material 1: Description of the microcosm systems***

To manipulate CO_2_ levels, temperature, and moisture, we developed a microcosm system using dry-bath cyclers and a custom-made CO_2_-dosage system. Falcon tubes (50 mL, Falcon, Greiner Bio-One, Frickenhausen, Germany) were filled with 30 g dry (80°C for 48 hr), sieved (2 cm mesh) soil (40% sand, 35% silt, 25% clay; Landerde, Ricoter, Aarberg, Switzerland). The natural soil microbiota was re-implemented to the soil as previously described (Hu et al., 2018). All falcon tubes were placed in dry bath cyclers (Digital Heating Cooling Drybath, Thermo Scientific, Fisher Scientific AG, Reinach, Switzerland) equipped with heating blocks that can accommodated up to nine falcon tubes. A CO_2_ mixing and distribution system was designed to continuously mix CO_2_ ambient air, measure the CO_2_ concentration of the mixture, and distribute it to different channels. Mixing CO_2_ and air was achieved using an air compressor (Prematic AG, Affeltrangen, Switzerland) coupled to two mass-flow-controllers (for CO_2_: Bronkhorst El-Flow Select F-200CV (0.6 mL.min^-1^), Ruurlo, Netherlands; and for air: CKD FCM-0010AI (0-10 L.min^-1^), CKD Corporation, Aichi, 485-8551, Japan). Ambient air from outside the building was used for mixing, therefore no CO_2_ was added to mimic current conditions (=450 ppm ± 50 ppm). A concentration of 400 ppm CO_2_ (purity 100%, 54.6 L bottle, and pressure of output at 0.8 bars, Gümligen, Switzerland) was added to ambient air (=850 ppm ± 50 ppm) to reach expected RCP 8.5 scenarios. The resulting CO_2_: air mix was pushed through a filter of activated carbon (Camozzi, Warwickshire, United Kingdom) and split through valves (Needle Valve 2839-⅛, CKD, Aichi, 485-8551, Japan) into seven individual channels in a series. The first channel, referred thereafter as “CO_2_ measuring channel”, was connected to a CO_2_ sensor (Rotronic AG, Bassersdorf, Switzerland). The air flow circulated alternatively between the CO_2_ measuring channel (for 2 min) and experimental channels (for 2 min). The two minutes duration between experimental channels was sufficient to reach stable expected CO_2_ concentrations. In all assays, four experimental channels were used, alternating between ambient (channels 2 and 4) and CO_2_ enriched (channels 3 and 5) air. Therefore, the ambient or CO_2_-enriched air was distributed through all channels within 16 min. This cycle was repeated every 30 min (16 min air distribution followed by 14 min pause) over the course of the experiment. Each of the experimental channels had 12 outlets (One-Touch fittings-male Straight, Sang-A Pneumatic Co., Daegu, Korea). Polyurethane tubing (outer/inner diameter: 4/2.5 mm, length: 2 m, Sang-A Pneumatic Co., Daegu, Korea) was connected to the outlets and distributed the air to the Falcon tubes. The tubing was attached to the lids of the Falcon tubes using One-Touch fittings-male Elbow (Sang-A Pneumatic Co., Daegu, Korea). The flow rate sent through individual Falcon tubes was adjusted to 1 L.min^-1^. The outflow of the Falcon tubes was connected to a collection system, itself connected to the CO_2_ sensor to verify CO_2_ levels. The collected air was then released in the environment. The pneumatic and communication parts of the microcosms are described in the scheme below. Pneumatic parts (Solid lines) include a mass-flow controller for CO_2_, a mass-flow controller for the carrier gas, an activated carbon filter, and needle valves. Communication parts (Dashed lines) include a Rs232 module, an analog module, a relay module, and a main controller.


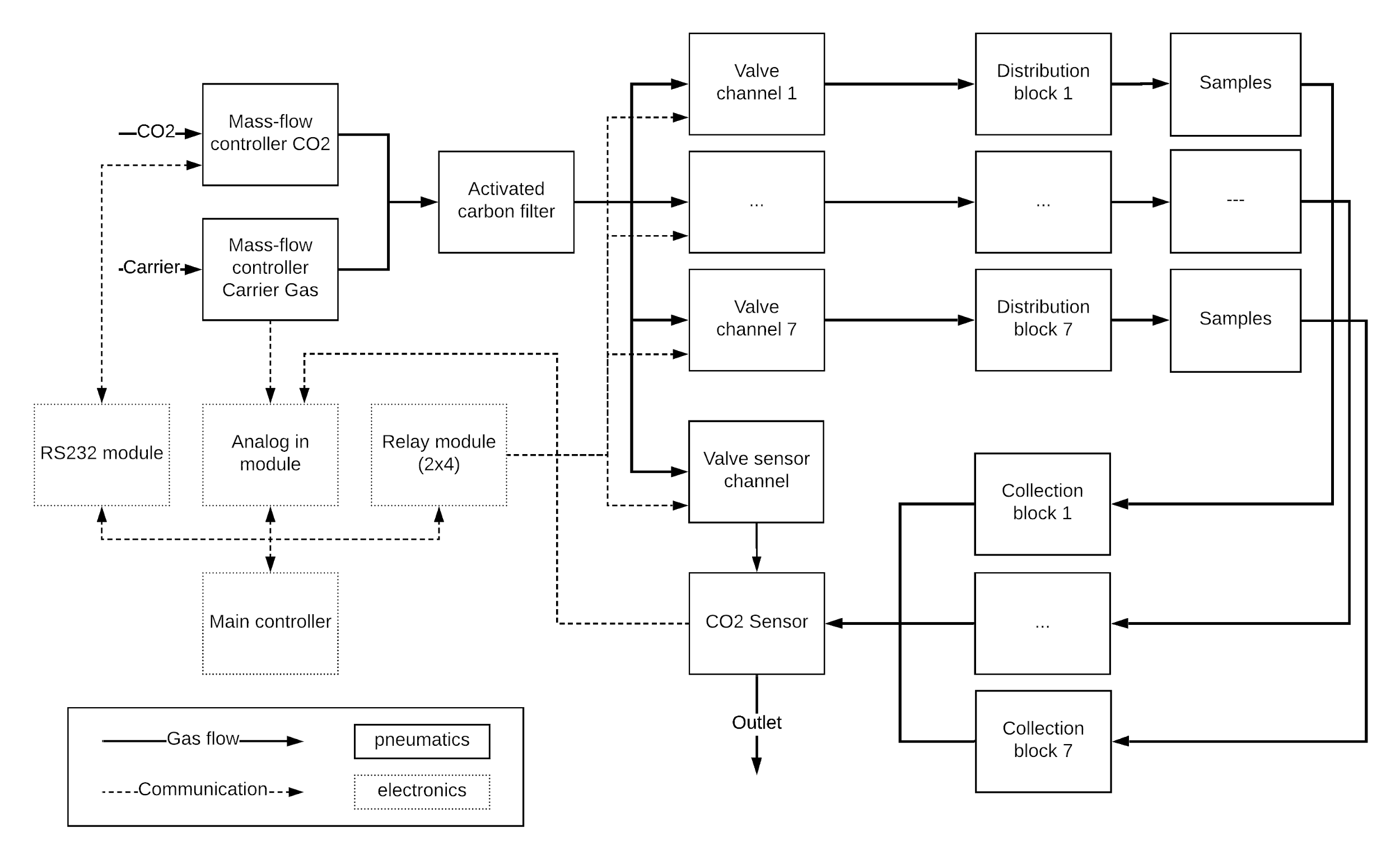

Supplement: Supplementary file 1 — Material S1 [file ECE3-11-4182-s004.docx]
