# Supplementary figures and images for: Natural enemies of herbivores maintain their biological control potential under short‐term exposure to future CO2, temperature, and precipitation patterns
Source: Ecol Evol. 2021 Mar 16;11(9):4182–92. doi: 10.1002/ece3.7314 (PMC8093683; doi:10.1002/ece3.7314)

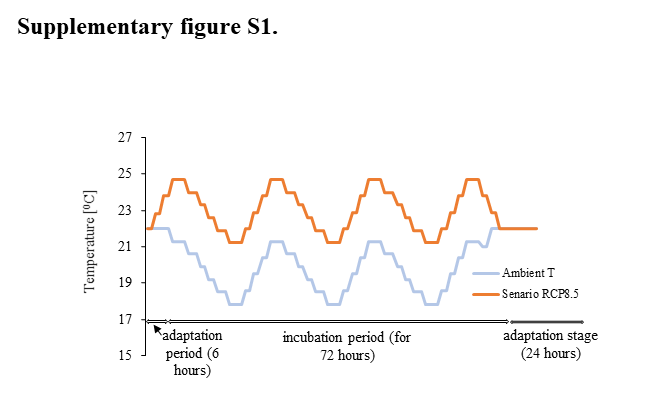

Supplement: Supplementary file 2 — Fig S1 [file ECE3-11-4182-s003.tif]
